# Epithelial to Mesenchymal Transition Relevant Subtypes with Distinct Prognosis and Responses to Chemo- or Immunotherapies in Osteosarcoma

**DOI:** 10.1155/2022/1377565

**Published:** 2022-07-04

**Authors:** Yang Zhou, Gai Li, Hu Li, Fuchong Lai, Pingguo Duan, Ming Cheng

**Affiliations:** Department of Orthopedics, The First Affiliated Hospital of Nanchang University, Nanchang, 330006 Jiangxi, China

## Abstract

**Objective:**

Currently, clinical classification of osteosarcoma cannot accurately predict the survival outcomes and responses to chemo- or immunotherapies. Our goal was to classify osteosarcoma patients into clinical/biological subtypes based on EMT molecules.

**Methods:**

This study retrospectively curated the RNA expression profiling of osteosarcoma patients from the TARGET and GSE21257 cohorts. Consensus clustering analyses were conducted in accordance with the expression profiling of prognostic EMT genes derived from univariate analyses. Immunological features were evaluated through immune cell infiltration, immune checkpoint expression, and activity of cancer immunity cycle. Drug sensitivity was estimated with the GDSC database. WGCNA approach was adopted to determine the EMT-derived genes. Following univariate analyses, a multivariate cox regression model was developed and externally verified. Predictive independency was evaluated with uni- and multivariate analyses. GSEA was presented to uncover relevant molecular mechanisms.

**Results:**

Prognostic EMT genes across osteosarcoma patients were stratified into distinct subtypes, namely, subtypes A and B. Patients in subtype B presented desirable prognosis, high immune activation, and enhanced sensitivity to cisplatin. Meanwhile, patients in subtype A were more sensitive to gemcitabine. In total, 86 EMT-derived hub genes were determined, and an EMT score was conducted for osteosarcoma prognosis. Following external verification, this EMT score was reliably and independently predictive of patients' survival outcomes. Additionally, it was positively linked to steroid biosynthesis.

**Conclusion:**

Overall, our findings proposed EMT-relevant molecular subtypes and signatures for predicting prognosis and therapeutic responses, contributing to personalized treatment and clinical implication for osteosarcoma.

## 1. Introduction

Osteosarcoma represents the most prevalent primary bone sarcomas, which originates from mesenchymal stem cell populations, with an annual incidence of 3-5 cases per million individuals [1–3]. It most often affects children, adolescents, and young adults, with aberrant bone growth areas [4]. The difficulty in biology research is in relation to the complexity of the osteosarcoma genome as well as remarkable biological discrepancy among osteosarcoma subtypes [5]. Hence, an in-depth understanding of osteosarcoma biology highlights the heterogeneity as well as uncovers the molecular abnormality that defines patient subpopulations. The standard treatment of osteosarcoma comprises surgical resection and chemotherapy [6]. Patients are usually resistant to conventional chemotherapy; meanwhile, high-dose chemotherapeutic agents cause severe side effects [7]. Hence, to improve the survival duration of osteosarcoma patients has been proven to be challenging.

Epithelial-mesenchymal transition (EMT) is a key process by which epithelial cells acquire mesenchymal characteristics [8]. Growing evidences demonstrate that EMT exerts a crucial role in stemness [9], metabolic reprogramming [10], immune evasion [11], and therapeutic resistance [12] for cancer cells. Osteosarcoma cells that have experienced EMT process will lose the cellular polarity and acquire aggressive and metastatic features. This process has been regarded as a crucial event for osteosarcoma metastases [13]. Previously, Yiqi et al. proposed an EMT-relevant model of osteosarcoma as a prognostic indicator through integrated multicohorts [14], indicative of the crucial prognostic implication of EMT during osteosarcoma progression. Limited evidences demonstrate that chemotherapy (cisplatin, etc.) resistance contributes to EMT activation in osteosarcoma cells [15, 16]. Recent research has presented that EMT genes are remarkably linked to immunity in osteosarcoma [17]. Tumor-associated macrophages trigger EMT in osteosarcoma through activation of COX-2/STAT3 signaling [18]. Thus, extensive research of EMT on clinical outcomes and therapeutic responses of osteosarcoma is urgently required. In our research, integrated genomic analyses were conducted for characterizing distinct EMT-relevant subtypes with prognostic and prediction implications in osteosarcoma.

## 2. Materials and Methods

### 2.1. Acquisition of Gene Expression Profiling

This study acquired the TARGET data matrix (https://ocg.cancer.gov/programs/target/data-matrix) comprising gene expression data and clinical data of 84 osteosarcoma patients, as the discovery cohort. Genome-wide gene expression profiling and prognostic information of 53 osteosarcoma patients were curated from the GSE21257 cohort in the Gene Expression Omnibus (GEO) repository (https://www.ncbi.nlm.nih.gov/gds/) [19], as the testing cohort. This cohort was on the basis of the platform of GPL10295 Illumina human-6 v2.0 expression beadchip. The gene expression series matrix files were directly retrieved, and the probe IDs were mapped to the gene symbols in accordance with the matched annotation files. Thereafter, expression values of multiple probes mapping to the same gene were averaged while probes mapping to multiple genes were eliminated. The gene set of EMT was curated from the Molecular Signatures Database (MSigDB), which was listed in Supplementary Table 1 [20].

### 2.2. Identification of Molecular Subtypes

Univariate analyses were presented for assessment of the association of EMT genes with osteosarcoma prognosis. Genes with *P* < 0.05 were determined for the subsequent analyses. *K*-means-based consensus clustering was carried out utilizing the ConsensusClusterPlus package on the basis of the transcriptome files of prognostic EMT genes [21]. Cumulative distribution function (CDF), delta area, and consensus matrix diagrams were conducted in accordance with default parameters. The number of clusters was determined in accordance with the following criteria: (i) the consistency within the clusters was relatively high; (ii) the coefficients of variation were relatively low; (iii) the area under the CDF curve was not remarkably elevated. The area under the CDF curves was utilized for defining the clustering number. Principal component analyses (PCA) were conducted for verifying the accuracy of this classification.

### 2.3. Gene Set Variation Analyses (GSVA)

Fifty hallmark pathways were acquired from the MSigDB, and the activity of above pathways was estimated with GSVA package in osteosarcoma specimens [22]. Limma package was adopted to compare the discrepancy in activity of hallmark pathways between subtypes [23].

### 2.4. Analyses of Immunological Features

The infiltration of tumor-infiltrating immune cells was estimated across osteosarcoma tissues utilizing the single-sample gene set enrichment analyses (ssGSEA) [24] and TIMER2 database [25]. The RNA expression of immune checkpoint molecules and HLA molecules was determined across osteosarcoma specimens. Cancer immunity cycle comprises release of cancer cell antigens (step 1), cancer antigen presentation (step 2), priming and activation (step 3), trafficking of immune cells to tumors (step 4), infiltration of immune cells into tumors (step 5), recognition of cancer cells by T cells (step 6), and killing of cancer cells (step 7) [26]. The activity of all steps was determined with ssGSEA approach in accordance with the transcriptome profiling [27]. The gene set of each step within the cancer immunity cycle was listed in Supplementary Table 2.

### 2.5. Estimation of Drug Sensitivity

Through adopting the pRRophetic algorithm [28], the half-maximal inhibitory concentration (IC50) values of cisplatin and gemcitabine were determined in osteosarcoma in accordance with the Genomics of Drug Sensitivity in Cancer (GDSC; http://www.cancerrxgene.org/) [29].

### 2.6. Weighted Gene Coexpression Network Analyses (WGNCA)

The data matrix of mRNA expression profiling in the TARGET cohort was analyzed utilizing the WGCNA package [30]. The first 25% genes with the largest variance were determined. For selecting a standard scale-free network, the sample hierarchical clustering approach was utilized for detecting and removing outlier specimens, followed by selection of the appropriate soft thresholding analyses. Thereafter, the adjacency matrix and topological overlap matrix (TOM) were established as well as the matched dissimilarity (1-TOM) was determined. Dynamic tree cutting methods were utilized for completing the gene tree and module clustering. Thereafter, the module characteristic genes were clustered as well as the highly similar modules were merged. The dissimilarity of the module eigengenes (ME) was determined with the moduleEigengenes function. The interactions of ME with EMT subtypes were analyzed with Pearson's correlation. Module membership (MM) represents the relevance of the expression profile to ME, while gene significance (GS) represents the correlation between the expression profiles and clinical traits. In this study, MM was calculated with Pearson's correlation of the expression profiling and ME. Meanwhile, GS was measured for evaluating the genes with EMT subtypes. Thereafter, the correlation between MM and gene signature was evaluated, and EMT-derived genes were determined.

### 2.7. Analysis of Hub Genes

Protein–protein interactions (PPIs) of genes in the lightcyan module were assessed through the Search Tool for the Retrieval of Interacting Genes/Proteins (STRING) online tool (http://string-db.org/) [31]. Hub genes in this PPI network were determined utilizing the Molecular Complex Detection (MCODE), a plug-in of Cytoscape software [32].

### 2.8. Functional Enrichment Analyses

Gene Ontology (GO) and Kyoto Encyclopedia of Genes and Genomes (KEGG) pathway enrichment analyses were presented utilizing the clusterProfiler package [33]. The parameter utilized in this package was default as well as the threshold for identifying the GO functions and KEGG pathways was set as *P* value < 0.05.

### 2.9. Gene Set Enrichment Analyses (GSEA)

For uncovering the difference in functional phenotypes between high- and low-risk subpopulations, GSEA was carried out. The “c5.all.v7.0.symbols.gmt” was utilized as a reference gene set. GSEA was implemented utilizing GSEA software. A nominal *P* < 0.05 was regarded as a significant enrichment.

### 2.10. Statistical Analyses

Statistical analyses were implemented utilizing R software (version 4.0.2). Kaplan-Meier survival curves were drawn among osteosarcoma cases, and survival difference was determined with log-rank test. Receiver operator characteristic (ROC) curves were presented with timeROC package. Comparison between two subgroups was conducted with student's *t* or Wilcoxon test. Two-sided *P* value < 0.05 was indicative of statistical significance.

## 3. Results

### 3.1. Characterization of Two EMT Molecular Subtypes in Osteosarcoma

**Figure 1:**
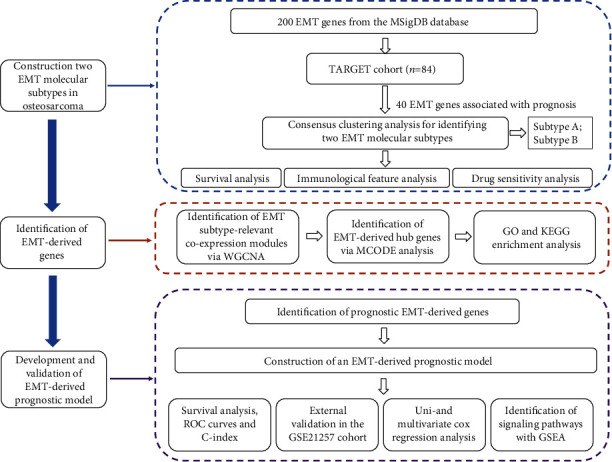
The flowchart of this study.

**Figure 2:**
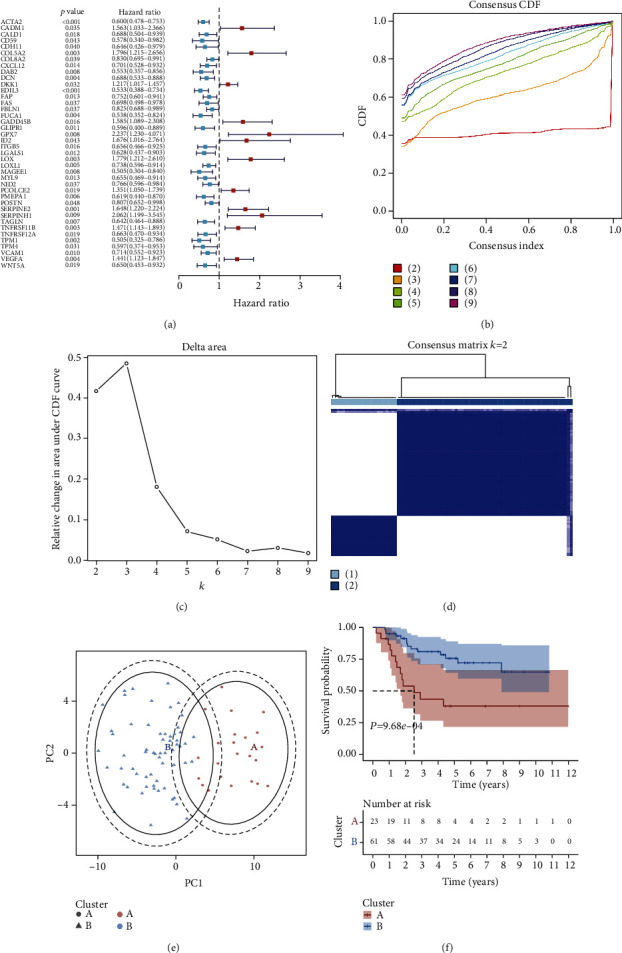
Characterization of two EMT molecular subtypes across osteosarcoma patients in the TARGET cohort. (a) Forest plots depict the univariate cox regression analyses results of EMT genes that were significantly linked to osteosarcoma prognosis with *P* < 0.05. Red indicates risk factor while blue represents protective factor. (b) CDF curves when *k* = 2 to 9. (c) The relative variations of the area under the CDF curves that *k* from 2 to 9. (d) The consensus clustering matrix at *k* = 2. (e) PCA plots visualizing the discrepancy in two clusters in accordance with the expression profiling of prognostic EMT genes. (f) Kaplan-Meier survival analyses for patients in two clusters.

Figure 1 illustrates the flowchart of this study. We curated 200 EMT genes from the MSigDB and analyzed their prognostic implication in osteosarcoma through univariate cox regression analyses. As a result, 40 EMT genes were remarkably linked to osteosarcoma prognosis (Figure 2(a); Table 1), in which 28 served as protective factors of osteosarcoma prognosis while the others served as risk factors. The consensus clustering of prognostic EMT genes was conducted to determine distinct EMT subtypes for osteosarcoma. For two categories, the area under the CDF curves began to stabilize (Figures 2(b) and 2(c)). Meanwhile, the consensus matrix was conducted for identifying the optimal number of subtypes. In Figure 2(d), the consensus matrix displayed a well-defined block structure when *k* = 2. Overall, two EMT molecular subtypes were conducted, namely, subtypes A and B. PCA results also fit into two clusters (Figure 2(e)). Survival analyses showed that subtype A presented poorer prognosis in comparison to subtype B (Figure 2(f)), indicative of the discrepancy in survival outcomes between subtypes.

### 3.2. Immunological Features and Drug Sensitivity of Two EMT Molecular Subtypes

**Figure 3:**
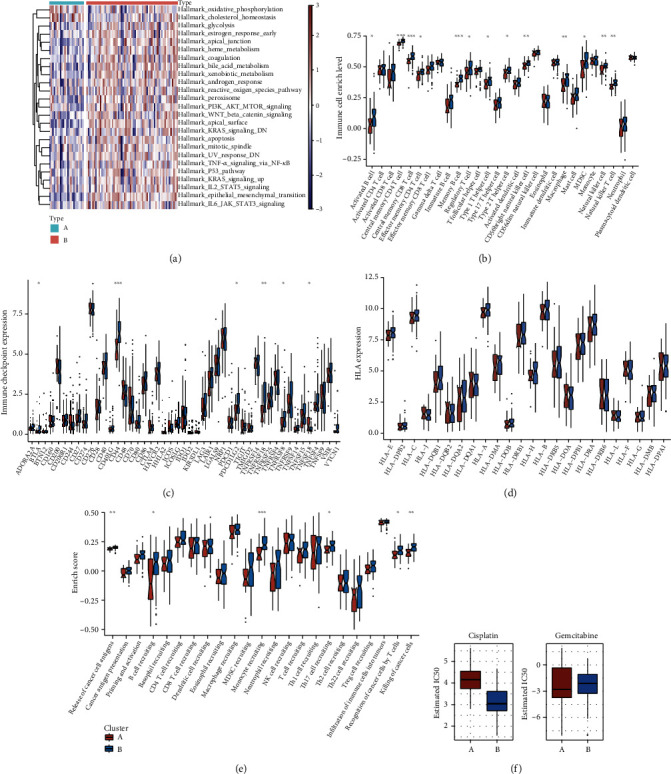
Immunological features and drug sensitivity of two EMT molecular subtypes. (a) Heat map depicts the activity of the 50 hallmark pathways in EMT subtypes A and B. (b) Boxplot shows the infiltrations of tumor-infiltrating immune populations in two subtypes. (c) Boxplot depicts the RNA expression of known immune checkpoint molecules in two subtypes. (d) Comparison of the RNA expression of HLA molecules in two subtypes. (e) Comparing the activities of all steps within cancer immunity cycle between subtypes. (f) Boxplot displays the sensitivity to cisplatin and gemcitabine in two subtypes. ∗*P* value < 0.05; ∗∗*P* value < 0.01; and ∗∗∗*P* value < 0.001.

The activity of the 50 hallmark pathways was estimated across osteosarcoma tissues utilizing the GSVA algorithm. We noted that the remarkable activation of stromal pathways (EMT, Wnt *β*-catenin signaling, etc.), immune pathways (IL6 JAK STAT3 signaling, etc.), and metabolism pathways (bile acid metabolism, heme metabolism, xenobiotic metabolism, etc.) in EMT subtype B than subtype A (Figure 3(a) and Supplementary Table 3). In Figure 3(b), ssGSEA approach showed that EMT subtype B presented remarkably enhanced immune cell infiltrations containing central memory CD4 and CD8 T cells, effector memory CD4 T cells, memory B cells, regulatory T cells, type 1 and 2 helper cells, CD55bright natural killer cells, macrophages, MDSCs, natural killer cells, and natural killer T cells than subtype A. Meanwhile, TIMER2 approach showed the lower infiltrations of B cells and the higher infiltration of CD4 T cells compared with subtype A (Supplementary Figure 1). Additionally, we noted that immune checkpoint molecules ADORA2A, CD44, PDCD1LG2, TNFRSF14, and TNFRSF8 were prominently activated in subtype B than A (Figure 3(c)). Nevertheless, we did not investigate the significant discrepancy in HLA molecule expression between subtypes (Figure 3(d)). The activity of cancer immunity cycle was also estimated in each osteosarcoma specimen. Compared with subtype A, release of cancer cell antigen, B cell/monocyte/Th17 cell recruiting, recognition of tumor cells through T cells, and killer of tumor cells presented remarkably enhanced activity in subtype B (Figure 3(e)). Overall, EMT subtype B possessed the features of immune activation. Also, subtype B displayed increased sensitivity to cisplatin while subtype A was more sensitive to gemcitabine (Figure 3(f)).

### 3.3. Establishment of EMT Subtype-Relevant Coexpression Modules

**Figure 4:**
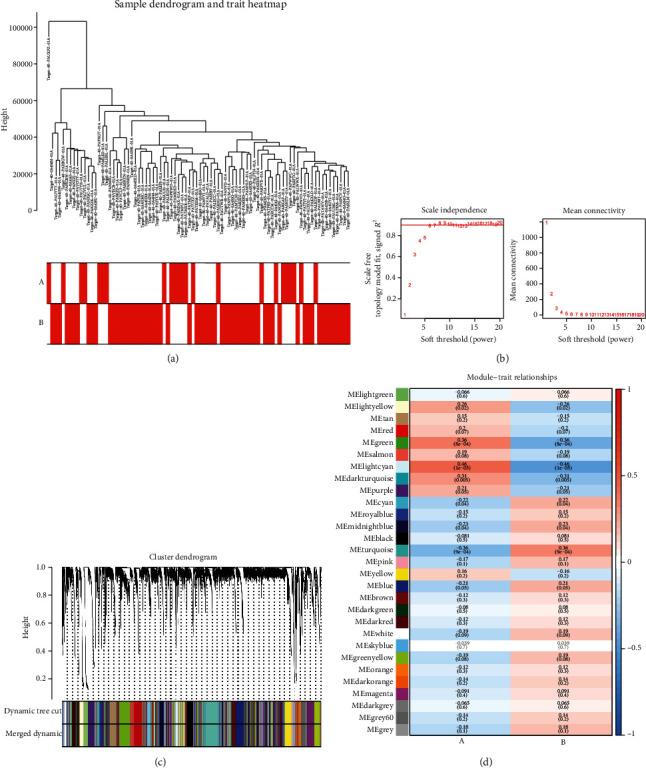
Establishment of EMT subtype-relevant coexpression modules in the TARGET cohort. (a) Hierarchical clustering dendrogram of osteosarcoma individuals as well as the matched EMT molecular subtypes. (b) Analyses of network topology and average network connectivity under diverse soft-threshold powers (*β*). The red line is indicative of a correlation coefficient of 0.85. (c) Clustering dendrogram with dissimilarity in accordance with topological overlapping, along with designed module colors. (d) The interaction network between modules and EMT molecular subtypes. Each column is indicative of EMT molecular subtype while each row is indicative of a coexpression module. The number in the rectangle displays the correlation coefficient and *P* value.

Considering the sensitivity of WGCNA to batch effects, this study firstly preprocessed the data of osteosarcoma individuals in EMT molecular subtypes A and B from the TARGET cohort. The first 25% genes with the largest variance were determined for subsequent analyses. Thereafter, the hclust function was adopted for confirming the batch effect removal as well as investigating whether there was any outlier. In Figure 4(a), no outlier sample was found. Because of the premise of WGCNA approach required to hypothesize that the network met the scale-free criteria, this study further screened the optimal soft thresholding value for making a coexpression network was more subject to a scale-free network. Through calculation of the scale-free topology fitting index, *R*^2^ value was up to 0.85 (Figure 4(b)), which validated the feasibility of WGCNA. A hierarchical clustering tree was retrieved through adopting hierarchical clustering analyses. Thereafter, in accordance with the dynamic tree cut approach, 29 distinct coexpression modules were assigned (Figure 4(c)).  Figure 4(d) demonstrated that the lightcyan module presented the strongest correlation to EMT molecular subtypes. The genes in this module deserved more exploration.

### 3.4. Exploration of EMT-Derived Genes and Their Relevant Biological Implication

**Figure 5:**
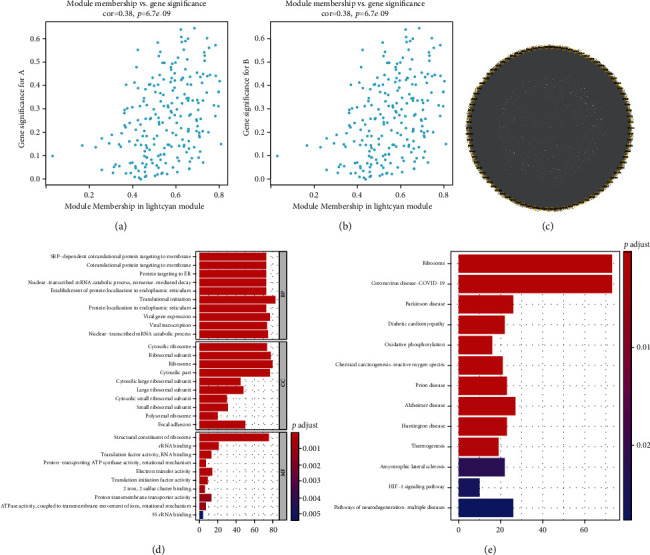
Exploration of EMT-derived genes and their relevant biological implication. (a) Scatter plots depict the interactions of module membership and gene significance for EMT subtype A. (b) Scatter plots display the interactions of module membership and gene significance for EMT subtype B. (c) The PPI network uncovers the EMT-derived hub genes through MCODE analyses. (d, e) GO and KEGG pathway enrichment analyses of EMT-derived hub genes.

Our further analyses uncovered that the genes in the lightcyan module were remarkably linked to two EMT molecular subtypes (Figures 5(a) and 5(b)), indicative of the genes in the lightcyan module as EMT-derived genes. Through MCODE analyses, we determined 86 EMT-derived hub genes, as depicted in Figure 5(c). Their relevant biological implication was explored in-depth. In Figure 5(d), the EMT-derived hub genes were remarkably linked to endoplasmic reticulum. Additionally, they were significantly enriched in ribosome, oxidative phosphorylation, chemical carcinogenesis-reactive oxygen species, thermogenesis, and HIF-1 signaling pathway (Figure 5(e)).

### 3.5. Development of an EMT-Derived Prognostic Model for Osteosarcoma

**Figure 6:**
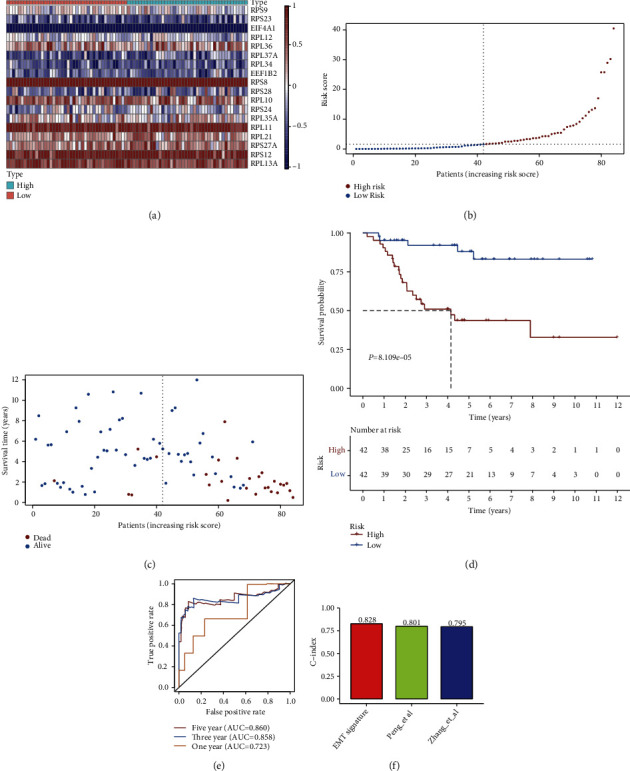
Development of EMT score for osteosarcoma prognosis in the TARGET cohort. (a) Heat map depicts the expression of each gene from EMT score in each osteosarcoma specimen. Red is indicative of upregulation; and blue is indicative of downregulation. (b) The distribution of risk score among osteosarcoma individuals. Vertical dotted line is indicative of the median value of EMT score. (c) The distribution of survival status of high- and low-risk subgroups. (d) Kaplan-Meier survival analyses for two subgroups. (e) ROC curves at one-, three-, and five-year survival in accordance with EMT score. (f) C-index of our EMT model and previously published two EMT models from Zhang et al. and Peng et al.

Our univariate cox regression analyses were indicative that 33 EMT-derived genes displayed significant interactions with osteosarcoma prognosis (Table 2). Above genes were input the multivariate cox regression model. In accordance with the expression and coefficient of candidate genes (Table 3), we developed an EMT-derived prognostic model for osteosarcoma, comprising RPS9, RPS23, EIF4A1, RPL12, RPL36, RPL37A, RPL34, EEF1B2, RPS8, RPS28, RPL10, RPS24, RPL35A, RPL11, RPL21, RPS27A, RPS12, and RPL13A. We noted the remarkable discrepancy in their expressions in high- than low-risk subpopulations (Figure 6(a)).  Figure 6(b) displayed the EMT score distribution across osteosarcoma patients. As EMT score elevated, dead cases were gradually increased (Figure 6(c)). Survival analyses demonstrated that high-risk cases were indicative of more unfavorable clinical outcomes in comparison to low-risk cases (Figure 6(d)). ROC curves were presented to assess the predictive performance of EMT score in osteosarcoma prognosis. In Figure 6(e), area under the curves (AUCs) at one-, three-, and five-year survival were separately 0.723, 0.858, and 0.860, proving that EMT score was capable of prediction of osteosarcoma prognosis. Compared with previously published two EMT models from Yiqi et al. [14] and Peng et al. [17], our EMT-derived prognostic model had higher C-index (0.828), indicating the prediction superiority of this model (Figure 6(f)).

### 3.6. External Verification of the Prognostic Value of EMT Score

**Figure 7:**
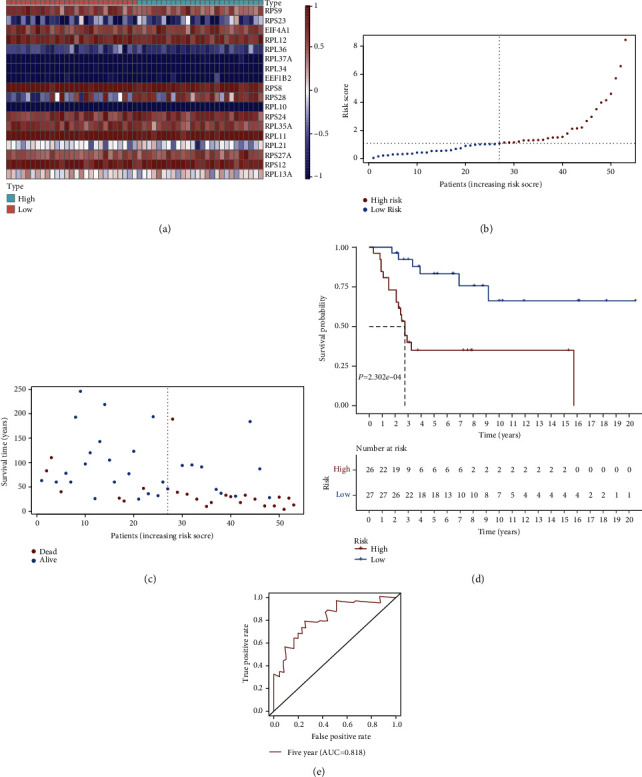
External verification of the prognostic value of EMT score in the GSE21257 cohort. (a) Heat map depicts the expression of each gene from EMT score in each osteosarcoma specimen. Red is indicative of upregulation while blue is indicative of downregulation. (b) The distribution of risk score among osteosarcoma individuals. Vertical dotted line is indicative of the mean value of EMT score. (c) The distribution of survival status of high- and low-risk subpopulations. (d) Kaplan-Meier survival analyses for two subpopulations. (e) ROC curves at five-year survival in accordance with EMT score.

The prognostic value of EMT score was further verified in the GSE21257 cohort.  Figure 7(a) depicted the expression patterns of each gene from EMT score across osteosarcoma specimens. We also visualized the distribution of EMT score in the GSE21257 cohort (Figure 7(b)). In accordance with the median value of EMT score, we stratified osteosarcoma individuals into high- as well as low-risk subpopulations. Additionally, we noted that there were relatively increased dead cases in high-risk subgroup (Figure 7(c)). As expected, high EMT score was remarkably linked to more unfavorable survival outcomes (Figure 7(d)). The AUC at five-year survival was 0.818, proving the excellent predictive performance of EMT score (Figure 7(e)).

### 3.7. Genes in EMT Score as Prognostic Indicators of Osteosarcoma

**Figure 8:**
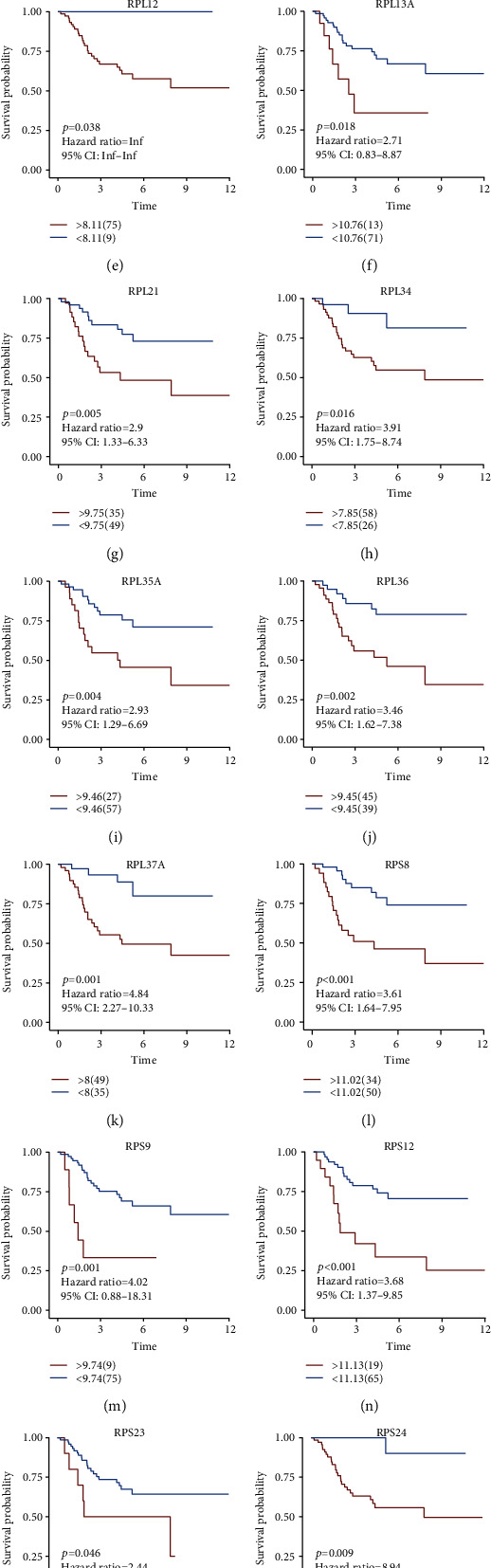
Association of genes in EMT score with osteosarcoma prognosis in the TARGET cohort. (a)–(r) Kaplan-Meier survival analyses for high and low expression subgroups of (a) EEF1B2, (b) EIF4A1, (c) RPL10, (d) RPL11, (e) RPL12, (f) RPL13A, (g) RPL21, (h) RPL34, (i) RPL35A, (j) RPL36, (k) RPL37A, (l) RPS8, (m) RPS9, (n) RPS12, (o) RPS23, (p) RPS24, (q) RPS27A, and (r) RPS28 in the TARGET cohort.

Further analyses were conducted for assessing the survival implication of each gene in EMT score in the TARGET cohort. As a result, high expression of EEF1B2, EIF4A1, RPL10, RPL11, RPL12, RPL13A, RPL21, RPL34, RPL35A, RPL36, RPL37A, RPS8, RPS9, RPS12, RPS23, RPS24, RPS27A, and RPS28 was prominently linked to more undesirable survival outcomes in comparison to their low expression (Figures 8(a)–8(r)).

### 3.8. EMT Score Is Independent of Clinicopathological Indicators for Osteosarcoma Prognosis

**Figure 9:**
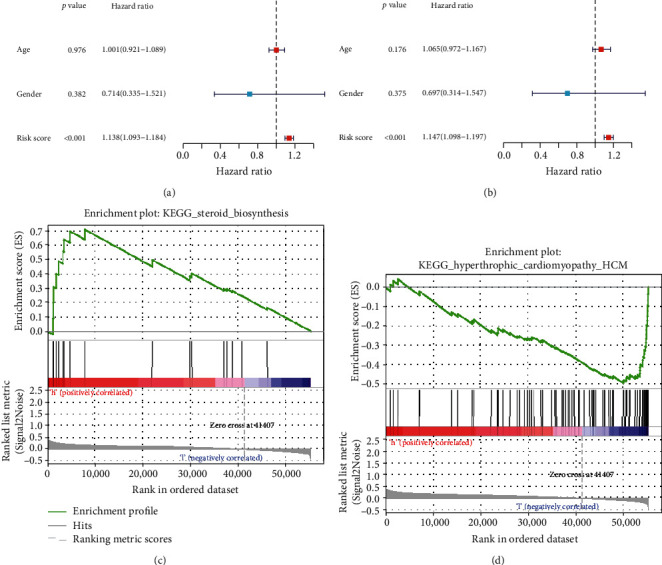
Evaluation of the independency of EMT score in osteosarcoma prognosis and its relevant signaling pathways. (a, b) Uni- and multivariate cox regression models for determining the independency of EMT score in estimating osteosarcoma survival outcomes in the TARGET cohort. (c, d) Signaling pathways that were remarkably linked to EMT score via GSEA.

In the TARGET cohort, our univariate cox regression analyses displayed that this EMT score was prominently linked to osteosarcoma survival outcomes (Figure 9(a)). Further multivariate analyses demonstrated that the EMT score was independently predictive of patients' prognosis (Figure 9(b)). For uncovering the underlying biological phenotypes between subpopulations, this research carried out GSEA. As a result, steroid biosynthesis was remarkably activated in high-risk subpopulation (Figure 9(c)), and hypertrophic cardiomyopathy displayed prominent activation in low-risk subpopulation (Figure 9(d)).

## 4. Discussion

Osteosarcoma presents diverse clinical courses and biological heterogeneity [34]. Hence, to reliably predict prognosis and therapeutic responses, it is critical for comprehensively investigating the molecular mechanisms. Our study determined forty prognostic EMT genes in osteosarcoma. Through consensus clustering approach, two EMT molecular subtypes were conducted. Especially, EMT subtype A presented poorer prognosis in comparison to subtype B, indicating that there was a remarkable discrepancy in survival outcomes between subtypes. Recently, immunotherapy is a promising treatment option against osteosarcoma, and it is crucial to improve the comprehending about the immune responses [3]. We evaluated the immunological features from multiple perspectives. GSVA demonstrated the activation of immune pathways (like IL6 JAK STAT3 signaling) in subtype B. Our ssGSEA revealed that subtype B displayed remarkably enhanced immune cell infiltrations containing central memory CD4 and CD8 T cells, effector memory CD4 T cells, memory B cells, regulatory T cells, type 1 and 2 helper cells, CD55bright natural killer cells, macrophages, MDSCs, natural killer cells, and natural killer T cells. In addition, subtype B had the features of increased expression of immune checkpoint molecules ADORA2A, CD44, PDCD1LG2, TNFRSF14, and TNFRSF8. Among steps within cancer immunity cycle, release of cancer cell antigen, B cell/monocyte/Th17 cell recruiting, recognition of tumor cells through T cells, and killer of tumor cells presented remarkably enhanced activity in subtype B. Hence, EMT subtype B was characterized by immune activation. Drug resistance severely hinders the improvement of survival rate of osteosarcoma patients [35]. Cisplatin, an alkylating drug, can form irreversible covalent bonds with DNA, which causes DNA strands to cross-link and break and missense mutation [36]. It is widely applied for osteosarcoma chemotherapy as well as cisplatin resistance is frequent across osteosarcoma individuals [37]. Our data demonstrated that EMT subtype B presented enhanced sensitivity to cisplatin. Gemcitabine represents the second cytidine analog developed following cytosine arabinoside [38]. Intriguingly, subtype A was more sensitive to gemcitabine.

Through WGCNA approach, we constructed 29 EMT subtype-relevant coexpression modules in the TARGET cohort. Especially, lightcyan module presented the strongest interactions with EMT molecular subtypes. Further, MCODE analyses determined 86 EMT-derived hub genes. Functional enrichment analyses unraveled the remarkable interactions of these EMT-derived hub genes with endoplasmic reticulum (ER), ribosome, oxidative phosphorylation, chemical carcinogenesis-reactive oxygen species, thermogenesis, and HIF-1 signaling pathway. For instance, the ER represents the central intracellular organelle of diverse cellular functions and endoplasmic reticulum stress response participates in osteosarcoma pathogenesis [39]. SENP1/HIF-1alpha regulates hypoxia-mediated EMT in osteosarcoma cells [40].

This study conducted an EMT-derived prognostic model for osteosarcoma in the TARGET cohort, comprising EEF1B2, EIF4A1, RPL10, RPL11, RPL12, RPL13A, RPL21, RPL34, RPL35A, RPL36, RPL37A, RPS8, RPS9, RPS12, RPS23, RPS24, RPS27A, and RPS28. ROC curves proved that the EMT score was capable of accurately predicting osteosarcoma individuals' clinical outcomes. Additionally, external verification proved the feasibility of this model in the GSE21257 cohort. Each gene in the EMT score was linked to undesirable prognosis of osteosarcoma individuals. Previously, mTOR inhibitor blunts the p53 response to nucleolar stress through modulating RPL11 and MDM2 expressions [41]. RPL34 upregulation is indicative of undesirable clinical outcomes in osteosarcoma as well as silencing RPL34 weakens osteosarcoma proliferation [42]. Suppressing RPS9 blunts osteosarcoma cell growth via inactivating MAPK signaling [43]. Nevertheless, the functions of most genes in osteosarcoma progression are lack of experimental evidences. Multivariate analyses suggested the independency of the EMT score in prediction of osteosarcoma outcomes. Further, GSEA results presented that steroid biosynthesis was remarkably activated in high-risk osteosarcoma individuals, indicating that steroid biosynthesis might affect osteosarcoma progression.

A few limitations of our study should be taken into consideration. First, further analyses are required for elucidating the molecular mechanisms underlying the impact of genes in the EMT-derived model on osteosarcoma through in-depth experiments. Second, more osteosarcoma patients can produce more convincing and accurate findings. Hence, abundant specimens will be included for improving our conclusions as well as more reliably illustrating the underlying mechanisms by which genes in the EMT-derived model affect osteosarcoma progression.

## 5. Conclusion

Collectively, this study adopted consensus clustering analyses for determining two EMT molecular subtypes in the TARGET cohort as well as uncovered the discrepancy in survival outcomes, immunological features, and drug sensitivity between subtypes. Through system biology-based WGCNA approach, we determined EMT-derived genes and conducted an EMT score for predicting osteosarcoma prognosis. To our knowledge, we firstly characterized the EMT-relevant subtypes for osteosarcoma. Additionally, the EMT score we proposed was externally verified in the external cohort. Overall, our findings might contribute to personalized treatment and be of much clinical implication for osteosarcoma.

## Figures and Tables

**Table 1:** Prognostic EMT genes of osteosarcoma in the TARGET cohort.

EMT genes	HR	HR.95L	HR.95H	*P* value
ACTA2	0.60007	0.478306	0.752831	1.02E-05
CADM1	1.563352	1.03282	2.366404	0.034631
CALD1	0.687834	0.503844	0.939013	0.018464
CD59	0.578201	0.340429	0.982043	0.042661
CDH11	0.646305	0.426496	0.979399	0.039579
COL5A2	1.796028	1.21462	2.655742	0.003344
COL8A2	0.830052	0.695262	0.990972	0.039373
CXCL12	0.701203	0.527838	0.931508	0.014302
DAB2	0.552834	0.356892	0.856351	0.007943
DCN	0.687972	0.532825	0.888294	0.004125
DKK1	1.217418	1.017358	1.456819	0.031726
EDIL3	0.533263	0.387603	0.733661	0.000112
FAP	0.751864	0.601025	0.940557	0.012547
FAS	0.698216	0.49836	0.978218	0.0368
FBLN1	0.825041	0.688457	0.988721	0.037269
FUCA1	0.538319	0.351718	0.82392	0.004346
GADD45B	1.585384	1.089054	2.307914	0.016163
GLIPR1	0.596184	0.399629	0.889413	0.011271
GPX7	2.237357	1.229706	4.0707	0.008362
ID2	1.676029	1.016465	2.763573	0.042973
ITGB5	0.656243	0.465517	0.925111	0.016205
LGALS1	0.628315	0.437404	0.902552	0.01191
LOX	1.778616	1.212237	2.609618	0.003241
LOXL1	0.738146	0.595854	0.914418	0.005456
MAGEE1	0.505129	0.30388	0.839659	0.008439
MYL9	0.654679	0.468967	0.913933	0.012821
NID2	0.765564	0.595752	0.983779	0.036818
PCOLCE2	1.351295	1.050002	1.739042	0.019333
PMEPA1	0.618542	0.439716	0.870092	0.005794
POSTN	0.806768	0.651984	0.998298	0.048195
SERPINE2	1.647594	1.220342	2.224429	0.001114
SERPINH1	2.061795	1.199287	3.544606	0.008863
TAGLN	0.641922	0.464091	0.887895	0.007398
TNFRSF11B	1.470617	1.142542	1.892898	0.002748
TNFRSF12A	0.662569	0.470071	0.933897	0.018749
TPM1	0.505179	0.324643	0.786111	0.002473
TPM4	0.597236	0.374292	0.952974	0.030618
VCAM1	0.71391	0.55234	0.922742	0.010049
VEGFA	1.440634	1.123444	1.847379	0.00401
WNT5A	0.64968	0.452996	0.93176	0.019071

**Table 2:** Prognostic EMT-derived genes in the TARGET cohort.

Genes	Hazard ratio	95% lower confidence interval	95% upper confidence interval	*P* value
RPS9	2.458541	1.15383	5.23857	0.01977
NACA	2.062809	1.072155	3.968812	0.030111
RPL31	1.680277	1.0025	2.816292	0.048903
RPS23	1.582119	1.016551	2.462348	0.042083
EIF4A1	1.937961	1.08181	3.471674	0.026127
RPL12	1.825521	1.014796	3.283939	0.044538
RPL36	1.802713	1.14667	2.834099	0.010684
RPL37A	1.925317	1.244403	2.978814	0.003262
RPL30	1.728499	1.013395	2.948215	0.044557
EEF1D	2.404885	1.289322	4.48567	0.005799
RPL34	1.591933	1.008351	2.513262	0.045971
EEF1B2	1.869724	1.109806	3.149981	0.018701
RPS19	2.010787	1.032395	3.916394	0.040005
RPS8	2.668652	1.469129	4.84757	0.001268
RPS28	1.782071	1.161261	2.734766	0.008189
RPL10	1.854512	1.061042	3.241354	0.030163
RPL17	1.641353	1.015359	2.653289	0.043159
RPS24	1.724982	1.073501	2.771832	0.024256
RPL35A	1.994541	1.117985	3.558361	0.01941
RPS29	1.749557	1.044543	2.930418	0.033539
RPS27	2.231872	1.311656	3.797681	0.003074
RPS6	2.021336	1.349611	3.02739	0.000639
RPL11	1.708468	1.002166	2.912551	0.049078
RPL10A	2.063278	1.076517	3.954529	0.029103
RPL21	2.199899	1.223921	3.95414	0.008404
RPS3	2.092433	1.247417	3.509873	0.005147
RPS27A	1.913786	1.113551	3.289098	0.018812
RPS7	1.752451	1.118447	2.745847	0.014344
RPS12	1.809168	1.044998	3.132147	0.034248
PABPC1	1.618195	1.005308	2.604728	0.047506
RPL3	2.218272	1.119994	4.393531	0.022315
RPL7	1.777043	1.066145	2.961965	0.027407
RPL13A	2.1936	1.139154	4.224085	0.01879

**Table 3:** Multivariate cox regression models of prognostic EMT-derived genes in the TARGET cohort.

Genes	Coefficient	Hazard ratio	95% lower confidence interval	95% upper confidence interval	*P* value
RPS9	4.023231	55.88136	4.964366	629.0281	0.001125
RPS23	-2.22729	0.107821	0.029177	0.398444	0.000838
EIF4A1	1.276326	3.58345	1.412445	9.091407	0.007211
RPL12	-1.6577	0.190577	0.047067	0.771666	0.020167
RPL36	1.628909	5.098309	1.267648	20.50471	0.021793
RPL37A	2.963511	19.36584	3.250895	115.3638	0.001135
RPL34	-1.15058	0.316453	0.088979	1.125463	0.075506
EEF1B2	-1.61765	0.198365	0.052239	0.753247	0.017492
RPS8	1.872409	6.503945	1.045504	40.4602	0.044678
RPS28	-0.96574	0.380701	0.125372	1.156025	0.088358
RPL10	1.556604	4.742688	1.641072	13.70634	0.004043
RPS24	1.05129	2.861339	0.805129	10.16888	0.104176
RPL35A	1.484525	4.41287	1.237412	15.73721	0.022118
RPL11	-2.61106	0.073457	0.007738	0.69735	0.022973
RPL21	1.896643	6.663489	1.460904	30.39358	0.014305
RPS27A	1.278613	3.591655	1.047995	12.3092	0.041895
RPS12	-0.85901	0.423581	0.148122	1.211305	0.109073
RPL13A	-3.5775	0.027945	0.002652	0.294432	0.002905

## Data Availability

The data used to support the findings of this study are included within the supplementary information files.

## Abbreviations

EMT: Epithelial-mesenchymal transition
GEO: Gene Expression Omnibus
MSigDB: Molecular Signatures Database
CDF: Cumulative distribution function
PCA: Principal component analyses
GSVA: Gene set variation analyses
ssGSEA: Single-sample gene set enrichment analysis
GDSC: Genomics of drug sensitivity in cancer
WGNCA: Weighted gene coexpression network analyses
TOM: Topological overlap matrix
ME: Module eigengenes
MM: Module membership
GS: Gene significance
PPI: Protein–protein interaction
STRING: Search Tool for the Retrieval of Interacting Genes/Proteins
MCODE: Molecular Complex Detection
GO: Gene Ontology
KEGG: Kyoto Encyclopedia of Genes and Genomes
GSEA: Gene set enrichment analyses
ROC: Receiver operator characteristic
AUCs: Area under the curves.

## Supplementary Materials

Supplementary 1 Supplementary Figure 1: the differences in immune cell infiltrations between two EMT subtypes using TIMER2 approach.

Supplementary 2 Supplementary Table 1: the information of EMT genes from the Molecular Signatures Database.

Supplementary 3 Supplementary Table 2: the gene set of each step within the cancer immunity cycle.

Supplementary 4 Supplementary Table 3: comparison of the activity of hallmark pathways in two EMT molecular subtypes.
