# Supplementary figures and images for: Epithelial to Mesenchymal Transition Relevant Subtypes with Distinct Prognosis and Responses to Chemo- or Immunotherapies in Osteosarcoma
Source: J Immunol Res. 2022 Jul 4;2022:1377565. doi: 10.1155/2022/1377565 (PMC9274235; doi:10.1155/2022/1377565)

Cluster A B

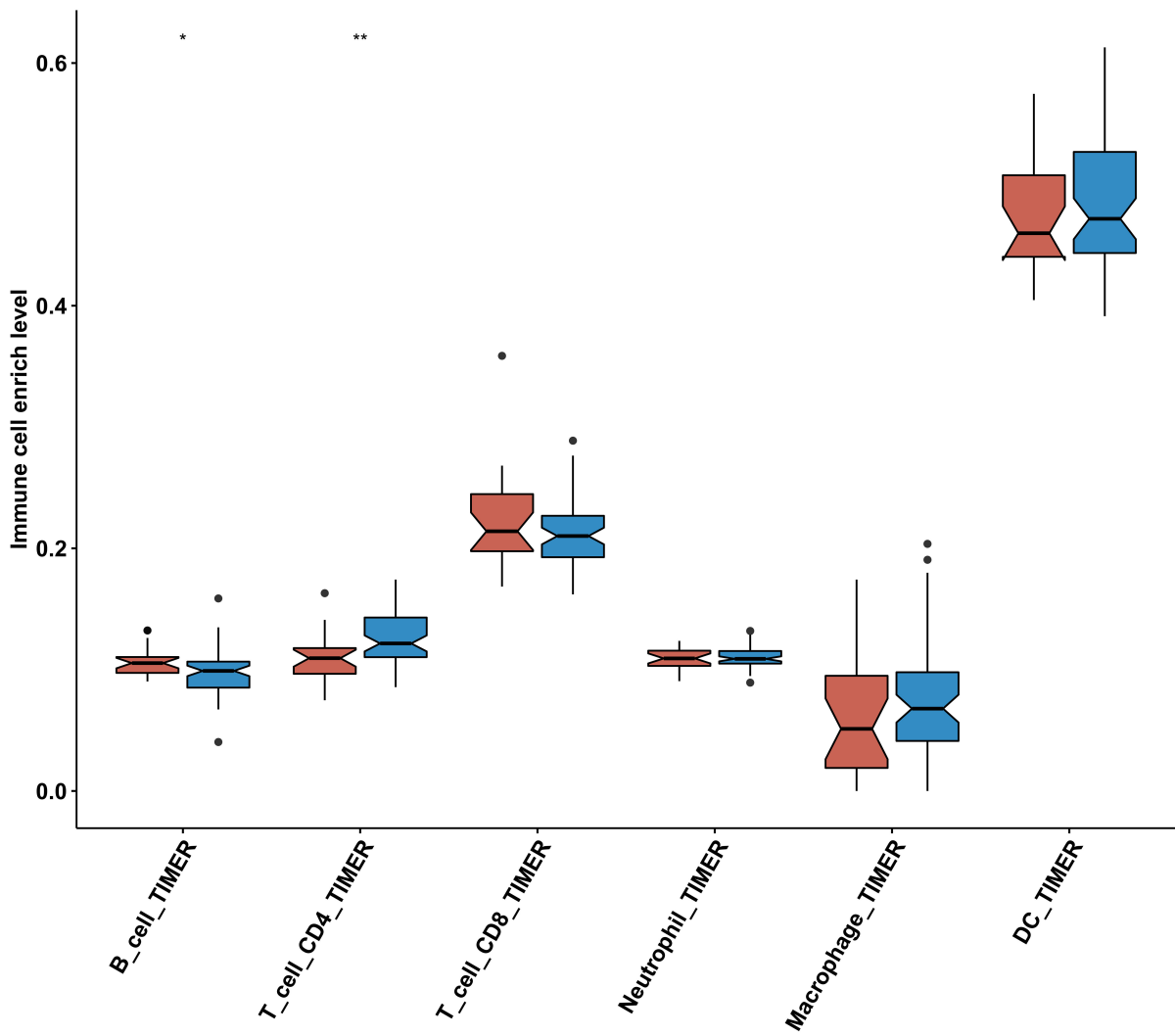

Supplement: Supplementary 1 — Supplementary Figure 1: the differences in immune cell infiltrations between two EMT subtypes using TIMER2 approach. [file 1377565.f1.pdf]
