# Supplementary material for: Epithelial to Mesenchymal Transition Relevant Subtypes with Distinct Prognosis and Responses to Chemo- or Immunotherapies in Osteosarcoma
Source: J Immunol Res. 2022 Jul 4;2022:1377565. doi: 10.1155/2022/1377565 (PMC9274235; doi:10.1155/2022/1377565)
Supplement: Supplementary 2 — Supplementary Table 1: the information of EMT genes from the Molecular Signatures Database. [file 1377565.f2.pdf]

Supplementary table 1. The information of EMT genes from the Molecular Signatures Database.

ABI3BP
ACTA2
ADAM12
ANPEP
APLP1
AREG
BASP1
BDNF
BGN
BMP1
CADM1
CALD1
CALU
CAP2
CAPG
CCN1
CCN2
CD44
CD59
CDH11
CDH2
CDH6
COL11A1
COL12A1
COL16A1
COL1A1
COL1A2
COL3A1
COL4A1
COL4A2
COL5A1
COL5A2
COL5A3
COL6A2
COL6A3
COL7A1
COL8A2
COLGALT1
COMP
COPA
CRLF1
CTHRC1
CXCL1
CXCL12
CXCL6
CXCL8
DAB2
DCN
DKK1
DPYSL3

DST
ECM1
ECM2
EDIL3
EFEMP2
ELN
EMP3
ENO2
FAP
FAS
FBLN1
FBLN2
FBLN5
FBN1
FBN2
FERMT2
FGF2
FLNA
FMOD
FN1
FOXC2
FSTL1
FSTL3
FUCA1
FZD8
GADD45A
GADD45B
GAS1
GEM
GJA1
GLIPR1
GPC1
GPX7
GREM1
HTRA1
ID2
IGFBP2
IGFBP3
IGFBP4
IL15
IL32
IL6
INHBA
ITGA2
ITGA5
ITGAV
ITGB1
ITGB3
ITGB5
JUN
LAMA1
LAMA2
LAMA3

LAMC1
LAMC2
LGALS1
LOX
LOXL1
LOXL2
LRP1
LRRC15
LUM
MAGEE1
MATN2
MATN3
MCM7
MEST
MFAP5
MGP
MMP1
MMP14
MMP2
MMP3
MSX1
MXRA5
MYL9
MYLK
NID2
NNMT
NOTCH2
NT5E
NTM
OXTR
P3H1
PCOLCE
PCOLCE2
PDGFRB
PDLIM4
PFN2
PLAUR
PLOD1
PLOD2
PLOD3
PMEPA1
PMP22
POSTN
PPIB
PRRX1
PRSS2
PTHLH
PTX3
PVR
QSOX1
RGS4
RHOB
SAT1

SCG2
SDC1
SDC4
SERPINE1
SERPINE2
SERPINH1
SFRP1
SFRP4
SGCB
SGCD
SGCG
SLC6A8
SLIT2
SLIT3
SNAI2
SNTB1
SPARC
SPOCK1
SPP1
TAGLN
TFPI2
TGFB1
TGFBI
TGFBR3
TGM2
THBS1
THBS2
THY1
TIMP1
TIMP3
TNC
TNFAIP3
TNFRSF11B
TNFRSF12A
TPM1
TPM2
TPM4
VCAM1
VCAN
VEGFA
VEGFC
VIM
WIPF1
WNT5A
