# Supplementary material for: Epithelial to Mesenchymal Transition Relevant Subtypes with Distinct Prognosis and Responses to Chemo- or Immunotherapies in Osteosarcoma
Source: J Immunol Res. 2022 Jul 4;2022:1377565. doi: 10.1155/2022/1377565 (PMC9274235; doi:10.1155/2022/1377565)
Supplement: Supplementary 3 — Supplementary Table 2: the gene set of each step within the cancer immunity cycle. [file 1377565.f3.pdf]

Supplementary table 2. The gene set of each step within the cancer immunity cycle.

GeneSymbol	Steps	Direction	ImmuneCellType
IL10	1	positive	Multiple
TGFB1	1	positive	Multiple
HMGB1	1	positive	Multiple
ANXA1	1	positive	Multiple
CALR	1	positive	Multiple
CXCL10	1	positive	Multiple
PDIA3	1	positive	Multiple
HSPA1A	1	positive	Multiple
HSPA1B	1	positive	Multiple
HSPA2	1	positive	Multiple
HSPA8	1	positive	Multiple
HSPA4	1	positive	Multiple
HSPA14	1	positive	Multiple
HSPA5	1	positive	Multiple
HSPA6	1	positive	Multiple
HSPA9	1	positive	Multiple
HSPA13	1	positive	Multiple
HSPA7	1	positive	Multiple
HSPA8	1	positive	Multiple
HSPA12A	1	positive	Multiple
HSPA12B	1	positive	Multiple
HSP90AA1	1	positive	Multiple
HSP90AB1	1	positive	Multiple
HSP90B1	1	positive	Multiple
IFNA2	1	positive	Multiple
IFNA1	1	positive	Multiple
IFNA13	1	positive	Multiple
IFNA6	1	positive	Multiple
IFNA21	1	positive	Multiple
IFNA4	1	positive	Multiple
IFNA8	1	positive	Multiple
IFNA5	1	positive	Multiple
IFNA7	1	positive	Multiple
IFNA14	1	positive	Multiple
IFNA16	1	positive	Multiple
IFNA10	1	positive	Multiple
IFNA17	1	positive	Multiple
IFNB1	1	positive	Multiple
IFNE	1	positive	Multiple
IFNW1	1	positive	Multiple
TNF	2	positive	Multiple
IL1A	2	positive	Multiple
IL1B	2	positive	Multiple
IFNA2	2	positive	Multiple
IFNA1	2	positive	Multiple
IFNA13	2	positive	Multiple
IFNA6	2	positive	Multiple
IFNA21	2	positive	Multiple
IFNA4	2	positive	Multiple

IFNA8	2	positive	Multiple
IFNA5	2	positive	Multiple
IFNA7	2	positive	Multiple
IFNA14	2	positive	Multiple
IFNA16	2	positive	Multiple
IFNA10	2	positive	Multiple
IFNA17	2	positive	Multiple
CD40LG	2	positive	Multiple
CD40	2	positive	Multiple
NT5C	2	positive	Multiple
HMGB1	2	positive	Multiple
TLR1	2	positive	Multiple
TLR2	2	positive	Multiple
TLR3	2	positive	Multiple
TLR4	2	positive	Multiple
TLR5	2	positive	Multiple
TLR6	2	positive	Multiple
TLR7	2	positive	Multiple
TLR8	2	positive	Multiple
TLR9	2	positive	Multiple
TLR10	2	positive	Multiple
HLAA	2	positive	Multiple
B2M	2	positive	Multiple
TAP1	2	positive	Multiple
IL10	2	negative	Multiple
IL4	2	negative	Multiple
IL13	2	negative	Multiple
CD3D	3	positive	Multiple
CD3E	3	positive	Multiple
CD3G	3	positive	Multiple
CD247	3	positive	Multiple
CD28	3	positive	Multiple
TNFRSF9	3	positive	Multiple
TNFSF9	3	positive	Multiple
TNFRSF4	3	positive	Multiple
TNFSF4	3	positive	Multiple
CD27	3	positive	Multiple
CD70	3	positive	Multiple
TNFRSF14	3	positive	Multiple
TNFSF14	3	positive	Multiple
CD40	3	positive	Multiple
CD40LG	3	positive	Multiple
TNFRSF18	3	positive	Multiple
TNFSF18	3	positive	Multiple
TNFRSF25	3	positive	Multiple
TNFSF15	3	positive	Multiple
TNFRSF8	3	positive	Multiple
TNFSF8	3	positive	Multiple
HAVCR1	3	positive	Multiple
TIMD4	3	positive	Multiple
SLAMF7	3	positive	Multiple
SLAMF6	3	positive	Multiple
SLAMF1	3	positive	Multiple

SLAMF9	3	positive	Multiple
SLAMF8	3	positive	Multiple
CD2	3	positive	Multiple
CD48	3	positive	Multiple
CD58	3	positive	Multiple
CD226	3	positive	Multiple
ICOS	3	positive	Multiple
ICOSLG	3	positive	Multiple
KLRK1	3	positive	Multiple
MICA	3	positive	Multiple
MICB	3	positive	Multiple
RAET1E	3	positive	Multiple
RAET1G	3	positive	Multiple
CRTAM	3	positive	Multiple
CADM1	3	positive	Multiple
CTLA4	3	negative	Multiple
PDCD1	3	negative	Multiple
PDCD1LG2	3	negative	Multiple
CD274	3	negative	Multiple
CD160	3	negative	Multiple
TNFRSF14	3	negative	Multiple
BTLA	3	negative	Multiple
VSIR	3	negative	Multiple
LAIR1	3	negative	Multiple
HAVCR1	3	negative	Multiple
HAVCR2	3	negative	Multiple
LGALS9	3	negative	Multiple
TIMD4	3	negative	Multiple
CD244	3	negative	Multiple
CD48	3	negative	Multiple
TIGIT	3	negative	Multiple
NECTIN3	3	negative	Multiple
LAG3	3	negative	Multiple
IL2	3	positive	Multiple
IL12A	3	positive	Multiple
IL12B	3	positive	Multiple
CXCR5	4	positive	B cell
CXCL13	4	positive	B cell
CCL24	4	positive	Basophil
CCL26	4	positive	Basophil
CCL19	4	positive	CD4 T cell
CX3CL1	4	positive	CD4 T cell
CXCL16	4	positive	CD4 T cell
CCR5	4	positive	CD8 T cell
CXCR3	4	positive	CD8 T cell
CXCL10	4	positive	CD8 T cell
CXCL9	4	positive	CD8 T cell
CCL20	4	positive	CD8 T cell
CXCL11	4	positive	CD8 T cell
CX3CL1	4	positive	CD8 T cell
CXCL16	4	positive	CD8 T cell
CCR7	4	positive	Dendritic cell
CCL3	4	positive	Dendritic cell

CCL4	4	positive	Dendritic cell
CCL5	4	positive	Dendritic cell
CCL21	4	positive	Dendritic cell
CCL11	4	positive	Eosinophil
CCL24	4	positive	Eosinophil
CCL26	4	positive	Eosinophil
CSF1	4	positive	Macrophage
CCL2	4	positive	Macrophage
CCL3	4	positive	Macrophage
CCL4	4	positive	Macrophage
CCL5	4	positive	Macrophage
CXCR2	4	positive	MDSC
CXCL5	4	positive	MDSC
CCL2	4	positive	Monocyte
CCL7	4	positive	Monocyte
CX3CL1	4	positive	Monocyte
CXCL1	4	positive	Neutrophil
CXCL2	4	positive	Neutrophil
CXCL3	4	positive	Neutrophil
CXCL8	4	positive	Neutrophil
CXCL6	4	positive	Neutrophil
CXCL5	4	positive	Neutrophil
CXCR3	4	positive	NK cell
CXCL10	4	positive	NK cell
CXCL9	4	positive	NK cell
CCL3	4	positive	NK cell
CCL4	4	positive	NK cell
CCL5	4	positive	NK cell
CXCL11	4	positive	NK cell
CX3CL1	4	positive	NK cell
CXCR5	4	positive	T cell
CCR7	4	positive	T cell
CXCL9	4	positive	T cell
CCL3	4	positive	T cell
CCL4	4	positive	T cell
CCL5	4	positive	T cell
CCL19	4	positive	T cell
CCL21	4	positive	T cell
CX3CL1	4	positive	T cell
CXCL13	4	positive	T cell
CXCR3	4	positive	TH1 cell
CXCL10	4	positive	TH1 cell
CXCL9	4	positive	TH1 cell
CXCL11	4	positive	TH1 cell
CCR6	4	positive	TH17 cell
CCL20	4	positive	TH17 cell
CXCL12	4	positive	TH17 cell
CXCR4	4	positive	TH17 cell
CCL1	4	positive	Th2 cell
CCL17	4	positive	Th2 cell
CCL22	4	positive	Th2 cell
CCR6	4	positive	TH22 cell
CCL20	4	positive	TH22 cell

CCR4	4	positive	Treg cell
CCR10	4	positive	Treg cell
CCL1	4	positive	Treg cell
CCL17	4	positive	Treg cell
CCL22	4	positive	Treg cell
CCL28	4	positive	Treg cell
STAT1	5	positive	T cell
IRF5	5	positive	T cell
KLF2	5	positive	T cell
ITGB2	5	positive	T cell
ICAM1	5	negative	T cell
EZH2	5	negative	T cell
DNMT1	5	negative	T cell
VEGFA	5	negative	T cell
EDNRB	5	negative	T cell
CD28	6	positive	Multiple
ICOS	6	positive	Multiple
ICOSLG	6	positive	Multiple
TNFRSF9	6	positive	Multiple
TNFSF9	6	positive	Multiple
CD27	6	positive	Multiple
CD70	6	positive	Multiple
TNFRSF4	6	positive	Multiple
TNFSF4	6	positive	Multiple
TNFSF14	6	positive	Multiple
CD40	6	positive	Multiple
CD40LG	6	positive	Multiple
HLAA	6	positive	Multiple
B2M	6	positive	Multiple
TAP1	6	positive	Multiple
BIRC5	6	positive	Multiple
MDM2	6	positive	Multiple
MAGEA4	6	positive	Multiple
TP53	6	positive	Multiple
PDCD1	6	negative	Multiple
PDCD1LG2	6	negative	Multiple
CD274	6	negative	Multiple
CTLA4	6	negative	Multiple
BTLA	6	negative	Multiple
VTCN1	6	negative	Multiple
IFNG	7	positive	Multiple
GZMB	7	positive	Multiple
PRF1	7	positive	Multiple
PDCD1	7	negative	Multiple
SMC3	7	negative	Multiple
VTCN1	7	negative	Multiple
HAVCR2	7	negative	Multiple
MICA	7	negative	Multiple
MICB	7	negative	Multiple
BTLA	7	negative	Multiple
VSIR	7	negative	Multiple
LAG3	7	negative	Multiple
IDO1	7	negative	Multiple

IDO2	7	negative	Multiple
ARG1	7	negative	Multiple
ARG2	7	negative	Multiple
NOS1	7	negative	Multiple
NOS2	7	negative	Multiple
NOS3	7	negative	Multiple
TGFB1	7	negative	Multiple
IL10	7	negative	Multiple
CCL28	7	negative	Multiple
CXCL12	7	negative	Multiple
CCL2	7	negative	Multiple
CXCL8	7	negative	Multiple
