# Supplementary material for: Epithelial to Mesenchymal Transition Relevant Subtypes with Distinct Prognosis and Responses to Chemo- or Immunotherapies in Osteosarcoma
Source: J Immunol Res. 2022 Jul 4;2022:1377565. doi: 10.1155/2022/1377565 (PMC9274235; doi:10.1155/2022/1377565)
Supplement: Supplementary 4 — Supplementary Table 3: comparison of the activity of hallmark pathways in two EMT molecular subtypes. [file 1377565.f4.pdf]

Supplementary table 3. Comparison of the activity of hallmark pathways in two EMT molecular subtypes.

Hallmark gene sets	logFC	t
Hallmark EMT	-0.0637	-7.77035
Hallmark Apical surface	-0.07485	-6.04392
Hallmark Apical junction	-0.03249	-4.89504
Hallmark Bile acid metabolism	-0.03967	-4.06134
Hallmark Heme metabolism	-0.0181	-3.7592
Hallmark Xenobiotic metabolism	-0.02485	-3.57791
Hallmark TNFα signaling via NFκB	-0.02906	-3.39164
Hallmark Wnt β-catenin signaling	-0.03231	-3.20319
Hallmark Androgen response	-0.01603	-3.0522
Hallmark Mitotic spindle	-0.01056	-3.04127
Hallmark Peroxisome	-0.02229	-3.01146
Hallmark UV response down	-0.02408	-2.95664
Hallmark KRAS signaling down	-0.03154	-2.80643
Hallmark Oxidative phosphorylation	0.008628	2.656973
Hallmark IL6 JAK STAT3 signaling	-0.02987	-2.6012
Hallmark Glycolysis	-0.01265	-2.57393
Hallmark KRAS signaling up	-0.02708	-2.53013
Hallmark P53 pathway	-0.01196	-2.4937
Hallmark IL2 STAT5 signaling	-0.02007	-2.38971
Hallmark PI3K AKT mTOR signaling	-0.01366	-2.35272
Hallmark Cholesterol homeostasis	0.015724	2.327394
Hallmark Estrogen response early	-0.02172	-2.11634
Hallmark Coagulation	-0.02072	-2.10474
Hallmark Reactive oxygen species pathway	-0.01676	-2.10445
Hallmark Apoptosis	-0.01727	-2.01012
Hallmark Angiogenesis	-0.02374	-1.92329
Hallmark mTORC1 signaling	0.006016	1.923159
Hallmark Pancreas beta cells	-0.03178	-1.7939
Hallmark Interferon alpha response	-0.02132	-1.78705
Hallmark Hypoxia	-0.01501	-1.77246
Hallmark Allograft rejection	-0.03222	-1.72432
Hallmark Inflammatory response	-0.03103	-1.67556
Hallmark Myogenesis	-0.02894	-1.53371
Hallmark Interferon gamma response	-0.02399	-1.40417
Hallmark Hedgehog signaling	-0.01862	-1.27624
Hallmark MYC targets V1	0.002062	1.120615
Hallmark Adipogenesis	-0.00419	-1.08714
Hallmark UV response up	-0.00519	-1.00149
Hallmark Spermatogenesis	0.00841	0.868797
Hallmark TGF beta signaling	-0.00426	-0.845
Hallmark Protein secretion	-0.00358	-0.83315
Hallmark Estrogen response late	-0.00956	-0.78128
Hallmark Unfolded protein response	0.001376	0.517418
Hallmark G2M checkpoint	-0.00198	-0.44327
Hallmark Notch signaling	-0.00383	-0.43917
Hallmark Fatty acid metabolism	-0.00228	-0.38593
Hallmark MYC targets V2	0.001548	0.294404
Hallmark E2F targets	-0.00129	-0.25802
Hallmark Complement	-0.00085	-0.08051
Hallmark DNA repair	-0.00012	-0.03963

P-value	Adjusted p-value
1.70E-11	8.51E-10
3.94E-08	9.85E-07
4.67E-06	7.78E-05
0.000109	0.00136
0.000313	0.003132
0.000577	0.004808
0.001059	0.007567
0.001918	0.011988
0.003037	0.01559
0.003138	0.01559
0.00343	0.01559
0.004032	0.016801
0.006219	0.023919
0.009428	0.033671
0.010968	0.036561
0.011802	0.03688
0.01326	0.039
0.014594	0.040539
0.019089	0.050235
0.020964	0.052409
0.02234	0.053189
0.037262	0.079822
0.038288	0.079822
0.038315	0.079822
0.047613	0.095227
0.057817	0.107098
0.057833	0.107098
0.076412	0.133213
0.07752	0.133213
0.079928	0.133213
0.088311	0.142437
0.097526	0.152384
0.128842	0.195216
0.163939	0.241086
0.205371	0.293387
0.265631	0.368932
0.280072	0.378476
0.319454	0.420335
0.387422	0.496476
0.400502	0.496476
0.40711	0.496476
0.436824	0.520028
0.606218	0.704905
0.658705	0.735177
0.661659	0.735177
0.700523	0.761438
0.769172	0.818269
0.797018	0.830227
0.936019	0.955122
0.968483	0.968483
